# Systems Medicine—Complexity Within, Simplicity Without

**DOI:** 10.1007/s41666-017-0002-9

**Published:** 2017-05-10

**Authors:** Richard Berlin, Russell Gruen, James Best

**Affiliations:** 1 Department of Computer Science, University of Illinois, Urbana, IL USA; 2 Nanyang Institute of Technology in Health and Medicine, Department of Surgery, Lee Kong Chian School of Medicine, Singapore, Singapore; 3 Lee Kong Chian School of Medicine, Singapore, Singapore

**Keywords:** Systems medicine, Complexity, Simplicity

## Abstract

This paper presents a brief history of Systems Theory, progresses to Systems Biology, and its relation to the more traditional investigative method of reductionism. The emergence of Systems Medicine represents the application of Systems Biology to disease and clinical issues. The challenges faced by this transition from Systems Biology to Systems Medicine are explained; the requirements of physicians at the bedside, caring for patients, as well as the place of human-human interaction and the needs of the patients are addressed. An organ-focused transition to Systems Medicine, rather than a genomic-, molecular-, or cell-based effort is emphasized. Organ focus represents a middle-out approach to ease this transition and to maximize the benefits of scientific discovery and clinical application. This method manages the perceptions of time and space, the massive amounts of human- and patient-related data, and the ensuing complexity of information.

## Introduction

Investigative biology received its early discipline from the thinking of philosophers such as Descartes (1596–1650) who taught that complex objects could be understood and studied by breaking the larger entity into smaller and smaller components. Following a related Newtonian method that identifies components and then the laws that rule them, this process is referred to as reductionism. There may be clusters of components within the larger design but reduction to the smallest is the pathway for understanding the whole. Much of science and biology in particular has followed this path. Reductionism has led to the recent unlocking of the genome, and the identification of innumerable biologic metabolic products and communicating molecules referred to as -omics (genomics, transcriptomics, proteomics, metabolomics)—the smallest particles and therefore the building blocks of an entire organism.

Now that the human genome has been sequenced and the reductionist results available, efforts concentrate on how to compose the knowledge of molecules into the networks and pathways of the body. The goal is to assemble the whole organism. As research working toward the entire human continues, mechanistic models are commonly used to describe the components in a linear fashion and imply cause and effect. At the center of this work lies the desire to associate models with genotype or metabolic function, ultimately to model phenotypic expression [1–3].

Toward the middle of the twentieth century, several investigators felt the need for more than reductionist methods; rather than reducing to the smallest components and then assembling, they described Systems Theory composed of an overlying hierarchy governing the interwoven levels of components below. Systems Theory was at home in biology where technological advances were moving rapidly, probing deeply into time and space. Systems Biology developed and now transitions to Systems Medicine, the application to disease and clinical problems [4–7].

However, Systems Theory has taken on many meanings during these transitions. Scientific investigation and the facility of medical providers at the bedside and of humans as patients themselves must be addressed and require clarity. While Systems Medicine deals with health and disease, complexity must be reduced and understanding, verification, and utility enriched [8–11].

### Systems Theory and Systems Biology

Progress in biology has made great strides in the past few decades. Technology advances the ability to measure shorter time intervals and nano-space consequently driving biology deeper to smallest components. Commonly in biology, components are assembled and become part of a “mechanistic” model. This model can be compared to a machine with fixed, defined parts that perform the same tasks and actions, the same operations each time. Such models, usually linear, help to convey an understanding of cause and effect, like a machine, the relations among the parts are assigned and fixed [2, 3]. Today the smallest components are genes, RNA, proteins, and metabolic products, (genomics, transcriptomics, proteomics, metabolomics—omics as collective) whether organized for metabolic processes or for regulatory and communication functions. Once defined, the process can be tracked from assembly to determination of biologic function and processes, ultimately portraying the pathway from genotype to phenotypic expression [12, 13].

Additional biologic explanation is required when the reductionist approach is inadequate, when components are missing or cannot be described satisfactorily [14]. Systems Theory is one such additional framework, a focus on the whole early in discovery to determine overlying principles that then guide the individual components (considered top down) rather than the reductionist bottom up approach to understanding. Schrodinger (1887–1961) sought more than reduced component parts and wrote the book *What is Life*? Publishing in 1943, Schrodinger realized the limitations of a mechanistic study in biology and the comparison of biologic discovery with machines. When he considered the redundancies of biological network structures and the signaling among cells, he was unable to apply the mechanistic metaphor; the study of life was beyond such limited tools for explanation [7].

Bertalanffy (1901–1972) wrote a more formal analysis, rejecting a purely reductionist and linear description, which became Systems Theory. Very much like Schrodinger seeking the secrets of life, Bertalanffy found obstacles to his progress in biology, problems that could not readily be solved through reductionist study. When he viewed the non-equilibrium state of an organism in its environment, Bertalanffy turned to unifying, non-linear principles running vertically through the whole rather than the more common reduced linear mechanistic description layered from the bottom up. After decades of work, he composed Systems Theory in the 1960s to address the limitations of reductionist thinking. He desired tools to study the whole, an entire Systems design that could be bounded and represented separately; the assembly of the smallest components would subsequently follow. Systems Theory became the study of Systems as a whole and from the “top down.” Overlying abstracted principles governed and explained the properties generated by the System, overseeing the basic components of the organism. Systems Theory required a hierarchical totality which designated irreversibly integrated relationships; one could not study a system in all its complexity only from the components since isolated units did not operate with one-direction causality and were insufficient to explain an entire living organism. For example, a bottom up description assembled from components would not explain behavior or adaptation [4, 15, 16].

Rosen (1934–1998) also rejected the machine metaphor and its associated mechanistic explanation. With the realization that functional components and pathways may be the result of the system itself, Rosen understood that models could be context dependent. Not every aspect of an organism was the result of the assembly of components and linear mechanisms; there were limitations which related directly to function and process. Reductionism seemed an unsatisfactory means to integrate the influence of environment and the emergence of properties from the totality of an organism. States and state change defined as reductionist structures of an organism were inadequate to explain the constraints or organism hierarchy. Other authors have agreed that reductionist experimentation has limitations [6].

However, Systems Theory required more than qualitative description. Rashevsky (1899–1972) added a quantitative mathematics emphasis. Rashevsky and Rosen worked on mathematical description in Systems Theory, demonstrating that there could be mathematical models in biology for cell division, diffusion, and integrated organism functions. In the belief that there could be best biologic design, Rashevsky and Rosen sought optimality rules, mathematics to describe organism subsystem function and energy expenditure [17].

In 1948, Wiener (1894–1964) wrote Cybernetics, a discussion of control and feedback, and Tustin (1899–1994) recognized that feedback itself was a fundamental principle supporting many processes of life. Feedback loops were readily implemented into models as negative feedback, often in loops with single or limited control measures or features; subsequent description did not stop at negative feedback—positive feedback, feed forward, adaptive and dynamic factors, and the discipline of control theory were added later in the century. Feedback is considered an example of a Systems Theory overlying principle, an example much as Bertalanffy had envisioned [18, 19].

From the vantage point of the physical world, Weiss (1898–1989) struggled with the conceptual integration of biologic knowledge. If biological systems exist in a world of thermodynamic laws, then such systems are dissipative and characterized by entropy. If the human body ages and is finally the product of prolonged deterioration, it is difficult to describe events mechanistically from reduced function of components. Entropy may be an overlying hierarchical principle in addition to feedback [6, 18].

A System is defined as a whole which can be bounded and self-contained. It has internal subsystems which are robust in the face of internal or external stimuli. There are characteristics of interdependency; a System is an indivisible unity with emergent properties, properties which are the result of the Complex System itself and not just of the individual components or units. When a subunit of a System is removed (hand, limb), it loses its emergent properties but the System may continue in an adjusted form [11, 20, 21].

Mesarovic originated the term Systems Biology by joining the concepts of Systems Theory and biologic investigation; the whole organism generated an environment that influenced the biologic component parts. The effects of culture and environment also have effects on an organism as it interacts with the external; these effects are not always predictable and thus trends in biologic evolution may face obstacles and take indirect, adaptive paths. Emergent, and at times evolutionary, properties arise through the influence that the entire organism has on component parts or that subunits and clusters of components have on each other. Emergence cannot be predicted. This is the description of a Complex System, multi-level subunits that are not fully independent and not fully linked: they are loosely associated through coupling principles. Homeostasis, in the view of Systems Theory, represents an emergent property of a Complex System, the balance managed by multi-level response and communication, ruled by principles inherent within the environment created by the whole organism itself [20–22].

Thus, Systems Biology is a comprehensive, quantitative, and dynamic study of organisms. The aspects of organisms may be non-linear, dynamic, and perhaps chaotic; they are inherently complex and non-stationary. There is a time variability property to biological processes; not all biological processes in a particular subunit or related to a particular function (on a small or large scale) proceed at the same speed. Biology often exhibits this time-scale separation whereby chemical reactions evolve at drastically different rates making models more difficult [23–26].

To this developing view of Systems Biology that the body is a total system of interwoven cells, tissues, and organs, connected by signals and managed by combinations of features, has been added the work on complex biological activity that combinations of communication channels within the body, at any and all levels are interleaved and function to maintain the body. This is no longer the mechanistic model of a heart pump being filled with enough blood, but a much more robust and involved definition. There are emergent properties such as homeostasis, behavior, and modularity; organisms live on the knife edge of stability. In Systems Theory, the concept of “holism” in biology refers to the overlying principles which guide the total, entire organism. “Holistic” consequently refers to such a Complex System and its hierarchy, capable of emergent properties [11, 27–30].

### Systems Biology—the Study of the Human

The study of physiology has been revived by taking the lead from Systems Biology. Dynamics and control became important areas of study reflecting the laboratory ability to recognize the metabolic and regulatory activators and the products of cell activity. Work concentrates on how elements interact, communicate, and function in relation to one another across subsystems and ordinary boundaries; this is different from the past when physiology tended to be more localized, descriptive, and isolated. Physiology maintains its emphasis on organ systems and adds the component relations of genome-cell-tissue as information becomes available, leading from molecule to organ and eventually to organism. Multi-level boundaries of cell and tissue are crossed as information is linked among subunits. The current interest in regulatory channels, communication methods, and cascading molecular networks adds to the more traditional organ centered and largely mechanistic knowledge. On the horizon are dynamic theory design and engineering control models [30–32].

Noble studied the heart, particularly rhythm function, extending prior work on cell action potential. He described the sodium and potassium ion channels in the heart and mathematically determined the sodium activation equations; these equations were used to model the generation of electrical current in the heart. Further study revealed that calcium channels were also intimately involved. This finding led to the understanding that the heart’s intrinsic pacemaker responded to the environment itself and not to one single molecular component; changes in voltage measured across cell membranes had an influence on ion flows and the resultant generated current. The more complex the results, the richer the understanding of cardiac pacemaker function. Noble realized that there were larger design features inherent within heart tissue that fostered cardiac rhythm and that function was ordered, not only the result of a bottom up assembly of components. The design influenced the process; there was a regulatory environment accompanied by boundary conditions for action potential and heartbeat [33].

Hunter spent time with Noble working on anatomical models of the heart and ventricular function. Subsequently, this work grew to include the entire human from molecule to organism, the Human Physiome Project (HPP). The HPP is a global network of research centers working on physiology and organ system function, from molecule to cell to organ and body taking inspiration from Systems Biology. The aim of the HPP is to work on particular physiologic problems, create models, and continue the experimentation, including additional subunits in the models as they are discovered. Computational modeling is central to the HPP meaning many disparate labs and many investigators working on personal areas of interest. Each contributes and it is hoped that eventually the whole will be generated. A future with in silico testing (computational predictive models) for individual patients as well as for populations is envisioned [34–36].

An important goal of the HPP is the Virtual Human Patient model, a vision to produce a computer graphic individual physiologic model for each human (as patient) and one of the vehicles for in silico work. The earlier work with Noble became the Cardiac Physiome centerpiece of the planned Virtual Human Patient. PhysioMap and a physiologic envelope for each patient at clinic visit are planned. In the future, one would visit a physician and find a computed Virtual Human Patient model available to track progress and help with suggested alternative treatments. The strategy includes models for drug testing and development, also through in silico clinical trials [35, 37–39].

However, some difficulties have arisen with the HPP; international funding and coordination remain issues. Many research facilities work in limited niche areas and do not communicate readily with the larger Physiome effort. There are individualized software applications which do not translate well: it is not uncommon to find difficulties when combining research on a particular organ or cellular system. A recent publication notes that 600 cell investigative software modules are available through the HPP with 400 more awaiting approval. Questions remain on how to merge this immense amount of data and how to construct consistent grand models involving dispersed datasets. It is not certain that the application of big data solutions alone can actually address these concerns [35, 40].

Another area in physiology which advances with the aid of Systems Biology is the study of -omics (genomics, transcriptomics, proteomics, metabolomics). With the aim to sort out innumerable protein and -omic biomarkers associated with heart disease, Bjornson is among many who worked on protein biomarker models. Bjornson’s goal is to match medical heart risk and the identification of the best predictive cluster of biomarkers. Heart disease risk would be identified by the isolation of patient risk and biomarker patterns, followed by incorporation within in silico simulation. However, -omics data is not well organized at present or available on an organ- or function-specific basis. Current electronic medical records do not provide ready access to such information on a patient by patient or population basis. There are many studies with conflicting results and thus associated risk is not easy to determine. Nevertheless, the belief remains that unique combinations of cardiac biomarkers will someday be predictive. The current reality is a flood of molecular fragments that faces a large gap to widespread bedside or patient use [41, 42].

The research of Vodovotz turns the fundamentals of Systems Biology to the study of human inflammatory cell and molecular defense. Working with models of trauma and the inflammatory response to sepsis (severe infection, often accompanied by multiple organ failure) or cancer, they model inflammation, traumatic injury, or circumstances where the body defense mechanisms fail. Inflammatory processes are understood to be much more than a temperature reading or white blood cell count: body defense response is described as intersecting cascades of inflammatory cells, biomarkers, and communicating proteins. Systems Biology has helped to illuminate the similarity between infection and cancer, an association not previously so apparent in cell communication or molecular involvement. Waves of inflammatory proteins or cytokines can be the body defense to infection; altered proportions of these same proteins can result in a dreaded opposite effect, cytokine storm. Similarly, the body defense guards against abnormal cells which might take hold as a cancer, while later, the same individual’s defense system allows clusters of malignant cells to form metastases in preferred locations. The regulatory molecular pathways and cascades of communicating proteins are much more complicated than initially thought but the broad insight of Systems Biology aids the way these investigations continue to uncover function and process—genes and even gene “mutations” do not work in isolation [43–48].

Systems Theory has been slow to bridge the gap to direct patient care; research delves to the depths of biological discovery and now links across subunits in biology studying regulatory and communication networks, yet translation directly to the provider for care or analysis or to the patient for deeper understanding remains distant. Holder and Clermont provide examples of labs that do address the goal to display with greater definition the dynamic physiologic signatures of critical illness. The obstacles of the most minute time and space measurement in the research lab are overcome, but bedside care still relies on linear heart beat and routine blood samples. Physiologic response monitoring through more frequent measurement, more frequent automatic analysis and synthesis, is required as well as help for the medical staff with the forthcoming masses of critical care data. As the molecular cascades of inflammatory response or cardiac cell injury are being uncovered in the lab, the dynamic physiologic signature of critical illness must be available at the bedside. These are important, vital objectives which remain insufficiently addressed [49–51].

Additional authors have remarked on the problems to assemble body subunits, to display on a medical record computer screen the expanse of molecular communication networks across biologic levels. Problems such as small world properties, circadian rhythms, and protein topology are assuming greater importance but challenge representation. Such laboratory descriptions rely on mathematics and computation, however, these dynamics remain quite far from clinical inclusion. There is no fixed scale at which Systems Biology is seen to operate—molecule, cell, or tissue—and a paucity of information on how to separate external versus internal organism changes [52–54].

Not only is there difficulty with the bedside presentation of molecular network cascades, organ risk biomarkers, or refined physiologic function, but there also lacks a true overlying hierarchical description of the human as complex system with emergent properties which requires overlying “holistic” principles as written by Mesarovic and Federoff. There are many components discovered but not the higher rules for order and assembly [20, 55].

## Systems Biology—the Search for Organizing Principles

The need for organizing principles in Systems Biology inspired by Schrodinger, Bertalanffy, Rosen, and Weiss remains unsatisfied. Schrodinger questioned what is life; living organisms are open systems which admit the transfer of energy between themselves and the environment. Life has an internal order disrupted by heat loss to the environment, an expression of entropy. He questioned why the few basic building blocks of inheritance have been maintained for millions of generations. There may be more efficient or more durable selection of characteristics, but that is not what has occurred. Generational stability is achieved in the face of an immeasurable number of molecular combinations not preferred. For Schrodinger scale mattered; constraints in time and space help determine a preference for order and perpetuation. Not unexpectedly, reductionism offers little guidance [56].

Contemporary writers Green, Mesarovic, and Wolkenhauer realize that, although the reductionist research method provides vast amounts of insight and understanding relating to the genome and -omic study, it leaves many basic issues unresolved. The data sets generated and the pathways described, now so many and cascading, require overarching views to simplify and bring further understanding to human biology. The search for organization continues. Not that the work on the genome or -omics or biomarkers is any less important; rather, one recognizes that crucial structure is still missing, an organizing hierarchical order. Complexity begets complexity without organization. A system of systems containing functions and processes requires principles regarding how they relate and interact. This is important for understanding, for communication, and to produce coherence as subunits and subsystems are assembled to represent a total organism. Organizing principles are needed [20, 57, 58].

Green questions whether biological networks are scale-free, whether measurements at different biologic levels are related in time or space. She supports the view that organisms are ordered in a modular and hierarchical fashion and that these structures have great influence on underlying function. Thus, cellular networks are robust in the face of external stimuli. To explain these properties as well as evolution, differentiation, creativity, and behavior, there must be a hierarchy of design. However, the temporal/spatial and guiding principles remain elusive. Without models to guide function and process, vast computation in biology leaves one with probabilities and partial explanations, more complexity rather than less. Constraint-based organization is required [2, 57, 58].

The multi-level nature of biology convinced Wolkenhauer to study biologic systems in search of organization. There are limitations to a molecular- or cellular-based approach. Larger subunits and organizing principles of the organism are required. If the hallmark of a disease is multi-level dysfunction, one must determine which overarching principles are involved. Examining disease, one must determine how the levels organize and respond, communicate, and react to what is happening above or below at a faster or slower pace, then transmit the disease exciting features from one level to another. It is uncertain whether these processes result from an external agent or alteration of inter-level molecular networks. The multi-level emergent property of homeostasis is disrupted. The organism as complex system, characterized by non-linearity requires simplified organizing principles to be understood. Wolkenhauer stresses a tissue-centered approach to evaluate the multi-level spatial and temporal relations although this might not be satisfactory for clinical application. He wonders whether a dynamic systems model might be most appropriate to model cellular processes and expression as tissue response [59, 60].

Noble turned from his work on the sodium/potassium channels in the heart and studied whether the genome itself carries all the information for organism formation. The enormity of the computation became apparent when he realized that the 25,000 or so human genes were likely building blocks whose instructions were actually determined by the environment in which they operated. His earlier work had demonstrated that much more was involved than sodium or potassium channels or ion concentrations to determine cardiac rhythm. He appreciated that guidance came from the larger internal environment and grander assembly. Echoing evolutionary biology and aspects of Systems Theory, evolutionary and functional paths need not be the most direct, or the most logical; other forces are operative and those principles must be uncovered [61–63].

To advance from Systems Biology to Systems Medicine and clinical application requires comprehensive and fundamental organizing principles seen from the top down that guide order. The human organism is a Complex System and forthcoming information should be organized for clarity and coherence. In contrast to a largely reductionist approach that produces disparate -omics, channels and networks or the work on the HPP which has generated so much non-consolidated software, organizing principles would provide guidance. Coherence and composition toward a greater totality, organization of common elements and characteristics, must drive the conception of the human organism. Such organizing principles are at least as important as specific genes.

A word of caution issues from Physics. There may be problems with “theories of everything”; there are areas where examination of time and space cross boundaries of scale that cannot be unified. Nevertheless, the limits of the reductionist method alone may have been reached and a Systems Theory structure for the human organism will be required [64–66].

### Systems Medicine—Systems Biology Addresses Disease and the Clinic

Hood remains at the forefront as Systems Biology transitions to Systems Medicine, taking a bottom up approach from genes and proteins to address disease, with application in the clinic and ultimately to health and wellness. Called P4 (medicine that is personal, participatory, predictive, preventive), Hood predicts that bedside analysis of 2500 genes and 50 organ systems will result by 2022 in “whole genome sequences, molecular profiling of diseased tissues, and periodic multi-analyte blood testing of biomarker panels for disease and wellness. The convergence of these practices will enable accurate prediction of disease susceptibility and early diagnosis of actionable preventive schema and personalized treatment regimens tailored to each individual [67].” A dataset of a billion elements will exist for each patient. This vision requires the composition of genetic information, the intersecting molecular processes of cells, and the signals and cascades of molecular networks. Similar to the HPP but without the emphasis on physiology and physiologic organ description and its vital sign functional measurement, Hood envisions true computations for each individual that are unique and accurate. Risk factors for diseases will be predicted and preventive measures, whether medication, activity-based or environmentally related will be possible. As needed, alteration of the individual’s genome will eventually be achievable so that disease predisposition can be eliminated; when possible, genome intervention to reverse abnormal bodily processes will be implemented. Culture, environmental factors, and social media will be encoded and included. The computation required is enormous, the participants many, and the patience profound. It is a “daunting” task [67–74].

In the words of Ayers, “The exponential development of highly advanced scientific and medical research analytical technologies throughout the past 30 years has arrived to the point where most (if not all) key molecular determinants deemed to affect human conditions and diseases can be scrutinized with great detail [75].” Collins agrees and in “a new initiative on precision medicine” emphasizes prevention and treatment strategies that take individual patient variability into account. The application of genomic and metabolomic knowledge, referred to as an extremely sound and comprehensive foundation of biologic knowledge, will drive medical description. Thus health predominates over disease. It becomes possible to predict later problems in individual health, to pre-emptively insert fragments into the genome, alter pathways, or design innovative treatments so that cancer does not occur and heart disease is avoided [76–79].

However, there are questions whether Systems Medicine when expressed as P4 medicine, without hierarchical and clear models for comprehension and communication, will have the success that is predicted. Organization is needed. Kirschner questions the possibility of treating disease as though it has a finite origin, likely molecular, as suggested by Hood, Ayers, and Collins. The spatio-temporal variability of biocomplexity seems overwhelming. Many factors, especially those beyond the strictly genomic must be taken into account; multi-level intersecting interventions operating at different scales in time and space will likely become known in the future when medical knowledge deciphers the cascades of regulatory and communicating networks. The workflows of healthcare professionals, local legal and financial constraints, customs, and regional environments will influence and separate the visionary finite proposal of Hood and Collins from the practical. Problems are too large to expect a computational result and resultant isolated, individual solutions [10].

There is disagreement that the Systems Medicine as defined by Hood and Collins is even computationally possible. Noble questions whether the computation is becoming so involved with the discovery of ever new pathways and cascades of intersecting markers that the mathematics is beyond calculation. Others believe that one cannot really assemble the whole from a reductionist assembly of components. There is a need for organizing principles, for abstractions which leave out elements but render a system understandable and workable. Wolkenhauer notes the need for further statistical tools to uncover the secrets of multi-level internal structure [2, 59, 62].

Kueper agrees that such understanding of the human will not be possible from computational models, assembled from the molecules and cells, alone. Disease is an abnormal response of molecular processes. It is complex and not isolated; it is multi-level and cross communicating. Networks are involved; asynchrony of messaging components and non-linear processes respond to abnormality and are beyond description until underlying hierarchical principles and subunits are evaluated. The question arises of the ensuing vast computation required to describe accurately the multi-level and multi-dimensional requirements of homeostasis. Will such computation be accurate, will it really interweave as presently conceived, and will the entire description of the human be coherent are questions that remain unanswered. The mathematics and computation for these next stages are yet to be clearly determined [11, 60, 80].

There are no privileged levels of causation. Noble suggests a theory of biological relativity and downward causation as vital for understanding. Similar to the view of Federoff that emergent properties in Systems Medicine are dynamic, time-dependent interactions, Noble adheres to the traditional Systems Theory view demanding simplified hierarchical rules and design. These views echo Keller who questioned the foundation of genetic thinking and claimed there is insufficient evidence to support genetic determinism. A subsequent explanation is required for multi-level (gene-cell-tissue-organ-organism) communication and development [61, 62].

Vogt, responding to the description of P4 medicine by Hood and Collins and the related expectation of treatment for “health” and “wellness,” writes that the medicalization of health (going beyond treating “disease” to altering the genome to achieve health and wellness) raises additional questions. Vogt argues that the processes of such medicalization can hardly be described; there are no accepted standards, agreements, or measures. Massive computation is not necessarily the arbiter of health and wellness. The usage of the word holistic by Hood to describe this process (treating the “whole” person) is also problematic as it is at variance with the usage in Systems Theory. Merasovic and Federoff, echoing Bertalanffy and Schrodinger, assign holistic to the simplified organizing principles that guide understanding and communication of a Complex System such as a human. The usage of holistic associated with Hood’s Systems Medics and P4 medicine is different. This usage is far removed from the drive for hierarchical principles overlying a complex organism [28, 81].

The question of life and the human is not merely the assembly of probabilities from a vast database. It seems quite unlikely that technology alone will produce the emergent properties of a Complex System; emotion, behavior, creativity, and individuality particularly in response to injury and disease are not likely to be described. Human elements must be maintained [81].

Additional investigators have discussed the association of biological discovery and the fundamentals of hierarchy, causation, and explanation in biology. For discovery in biology to have utility, especially at the bedside, there must be relation to change in the patient state, to causation as understood albeit weakly. Love writes of the importance of top down causation; hierarchy is important to explain causation and emergent properties. Relating to multi-level biologic designs, Butterfield notes that causation and dynamics may differ at subsequent levels; emergence may be independent of reductionism on a variety of scales. Auletta believes top down causation in the setting of boundary conditions throughout a dynamic system is best explained by hierarchy and modular subunits. Finally, Kleinberg describes the difficulties of causation in medicine supported by single (token) incidents. Reviews of collections of patients, clinical trials, and subsequent verification are to be preferred. Causation is unlikely to be a successful characteristic if its primary origin is computation, unique to an individual, without associated hierarchy. The medicalization of health appears likely to be relegated to the very distant future [82–87].

## Situation Awareness

Endsley was among the very first to investigate situation awareness: the mental models that represent the perceived state of an individual’s environment. Situation Awareness is distinct from decision making itself; it is the comprehension of time and surroundings and projection into the future. In medicine, it is understood that awareness is critical to making correct decisions, especially in stressful or critical situations. It is an internal mental model [88, 89].

For Endsley, memory is central to situation awareness. Thus, history, comprehension, tracking, fatigue, communication, and integration are all important features; in medicine, these elements should not be left entirely to the responsibility of each individual, provider, or patient. The border of situation awareness and medical diagnosis, information exchange and data integration, becomes blurred. Endsley added, “Unfortunately, in the face of this torrent of data, many operators may be even less informed than ever before.” As larger medical teams of individuals become involved in the care of any one patient and as medical problems become more difficult and the processes more complex, the individual and shared situation awareness will become more strained and difficult to manage [88].

In environments of high stress that are information-intensive and time-constrained, situation awareness has an ever-increasing influence on decision making. In medicine, this means diagnosis [88]. The study of situation awareness itself has moved beyond studying only a single, isolated individual to examine distributed situation awareness and team-related situation awareness. Whether a team in an operating room, emergency room, or ICU, awareness is much more than a single diagnosis or the timing for an intravenous medication. Each member of a team must master their own duties, be aware of others, and expect to manage slips and lapses as important information is passed. More difficult is a distributed example such as transport of a patient from a remote medical facility to a regional center. The ambulance crew, the waiting intensive care team, and associated specialists must all be aware of what is occurring, what is expected and how to participate or face the unanticipated [90–92].

Drews studied the ICU and listed error-producing conditions for the ICU staff. Among these are devices that have low signal to noise ratios (hard to discriminate the noise), false sensor readings, cumbersome interfaces which do not map to the task at hand, and shortage of time. The coming deluge of genomic and metabolomic values will not make the tasks any easier. As Drews emphasized, the ICU is a difficult environment when its design relies heavily on “single-sensor-single-indicator” display. Alarm fatigue is well known. Nurses in an ICU perform most of the patient-focused activities, much as in a clinic or through a home health visiting program. Nurses rely on trend determination; technology should be enlisted to reduce trend tracking as a largely human mental function. Rather than rely on the individual physician or nurse to recall past lab values, chart patterns, or monitor values, it is time for engineering to design situation awareness aids to help healthcare provider teams track, predict, and compare vast amounts of information. If the science is correct, the diagnoses will be supported by precision analytics. In such a world, situation awareness rises all the more in importance [93–96].

Singh investigated medical errors and compared medicine with aviation. Diagnostic errors were the primary concern. Rather than focus on the individual who “committed” the error, he wisely turned his interest to define systems and support such that errors were rapidly recognized and useful feedback to the individual and the team was encouraged. The emphasis was on structures which supported practice rather than checklist type sheets which are rapidly out of date after completion. Singh emphasizes an engineering approach providing the means to correct discontinuities of care and generic communication and data integration problems. He writes that tasks and cognition must be supported by systems and awareness [97].

Time measurement is a constraint for humans in medicine. Humans are poor at estimating the passage of time, a common problem under stress. Cooper adds that the factors of noise, recall, and fatigue come into play affecting the entire team. Bolstad agrees that situation awareness is not a simple construct; rather situation awareness is a complex process in which multiple features should be considered. Complexity further increases when contributing factors to individual situation awareness must include shared situation awareness, the influence that individuals have on each other and on the environment. Situation awareness is not necessarily a sequence; rather, it can be a series of unconnected incidents or intersecting but independent cycles. The action and participation are continuous but not necessarily linear or logical, true whether in ICU or clinic [98, 99].

Studies demonstrate that the human mind can manage 4–5 variables at a time. Additional variables and the human mind cannot readily comprehend; confusion and dissociation results. Thus, medical information, physiology, and patient characteristics must be organized into clusters and united through models and principles [100, 101].

The information presentation, mental models, and problems that humans face determining the rates of variable change and pattern recognition limit awareness. Comorbidity, asynchrony, drug interactions, emergent response, and cascades of inflammatory markers characterize urgent medical management and physician-patient communication. As genomics and metabolomics and the benefits of the HPP come to the bedside or the home console, situation awareness must be studied to determine the best methods for humans to assimilate the vast amounts of information. Situation awareness is often considered to be reactive; it must become strategic and prepared. The human becomes informed, ready to perform, and able to accept the unfolding situation and forthcoming data [102].

A massive computational P4 model, as described by Hood or Collins, that generates a black box-type point genomic or metabolomic “solution” to a suspected health or wellness problem does not contribute to a mental model, or to a conclusion of causation. Such a solution inhibits human-human interaction when lacking common grounds for expression. Computational probabilities and the likelihood of later health or disease may lead to confusion rather than clarity; the examples of genetic testing, cancer markers, and cardiac risk factors are well known. Clarity is not often the result of complex computation or of a billion data points. In addition, the lack of validity of predictions that are solitary and have minimal means to track and verify results reduces the ability of P4 medicine to enhance situation awareness [103–106].

Karwowski writes that situation awareness has become so complex, one needs to consider the human-machine interface as part of the problem. The human may respond in a complex, adaptive, perhaps unpredictable manner. Supplying the information to the human in raw form, the approach for the last many decades, is no longer adequate. There is need to design the human-machine interface taking into consideration the human response. This process does not minimize the role of the human but optimizes the flow of information. It can be impossible to predict either the response of the human or the response of the machine. Information must be organized to further mental models and improve situation awareness. The patient’s experience in the hospital and at home is an equally important consideration. Common models and descriptive communication are effective tools. Causal reasoning is intimately linked with mental models and provides all the more reason that fundamental forthcoming medical and health information must be properly presented. Our ability to generate information about a patient has outstripped our ability to interpret it. This is particularly true when frequent time measurements, minute spatial definitions, and overwhelming masses of information confront the human mind [107, 108].

The human is the integral part of an increasingly complex adaptive environment of medicine, disease, patient care, and of health; mental models assume ever greater importance. New methodologies are sought to generalize cellular functions into concepts of tissue organization to improve understanding. Mental models are closely linked with causative thinking and the centerpiece of situation awareness; without appropriate models and hierarchical design, there is little likelihood of cognitive modeling and certainly a quite limited ability to recognize patterns intuitively. Levy and Bechtel emphasize that these concerns should be addressed: abstractions and models are scattered along the path to organizing principles [2, 11, 109–113].

Perhaps, much can be learned about the human-information-machine interface and situation awareness from aviation and cockpit design. Similar to other advanced engineered systems [114, 115], Mouthaan describes the F-16 intelligent cockpit as an environment. The cockpit works hand in hand with the pilot; it tries to sense that the pilot is landing the jet, giving hints as to pitch angle and the relation of the nose of the aircraft to the ground. Skaff describes the F-35 cockpit in which the pilot returns to the role of tactician. The guiding principle of the F-35 design is that computers should do what they do best, and pilots should perform where they are most capable. The cockpit works with the pilot and monitors response and performance [116–119].

The human-information-machine interface of Systems Medicine should function in a similar manner. The interface becomes flexible in both timing and content based on the challenge faced by the provider and the volume and strength of incoming information. The machine (computer, EHR, device) concentrates on what it does best—records time, follows innumerable variables, tracks multiple graphs simultaneously, varies the information display, and provides a historical record. Let the human make the decisions, take the responsibility, use intuition, and search for patterns and associations which are not immediately obvious and manage the human side of medicine [120, 121]. Find what works in the hospital, at the bedside, in the home and based on the environment of the cockpit of medical care, adapt the elements and rate of information absorption to the level of care, and the depth of understanding. Remove the immense needs to process information and track data from the shoulders of the human and let the physician fly the plane.

## Challenges: Does Complexity Lead to Systems Medicine? [8, 9]

1. Is there a need for Systems Medicine? Yes, the ongoing transition from inflated blood pressure cuffs and linear heart rate monitors to a world of profound understanding is vital. The barriers of time and space measurement are crossed and science must now assemble the components. This is a unique time in the history of medicine which requires the structure and organization of Systems Theory to become Systems Medicine.
2. What role does situation awareness play during the transition to Systems Medicine? When considering disease, health, and wellness, the human must be in the center. Human understanding as professionals under stress in the ICU or a patient suffering from illness must be primary. Human to human communication, meaning common ground for discussion and models for understanding, describes medicine. Professionals, patients, and the data interface must act in unison. Situation awareness is the environment of humans coping with illness and health.
3. What methods should be considered to reduce the complexity of the massive data forthcoming in disease and health? The computer/device screen is the common interface for humans and patient care. Current technology relies upon outdated screen presentation which is largely unchanged from the pre-computer era. At the bedside, physicians/nurses must collect massive amounts of information mentally, then sort and order this information. Medical success relies much too heavily on the human mind for these processes—design and computation is required to filter, sort, and display what is most important at the time in a manner most appropriate for human need. The presentation of information should fit human models of disease process and physiologic response so that the human can react. Situation awareness is enhanced. There must be a connection between cause and effect understanding in a world where much is not known for certain but critical decisions must nevertheless be made.
4. How should information be organized to reduce the complexity of information and of forthcoming discoveries? Traditional understanding in medicine revolves around physiology and its organ-based structure. Organ-based information should be viewed as “middle-out” and positioned as the central pillar around which additional knowledge is structured. The evidence of the overwhelming number of different Physiome-related cell models, the difficult graphs in inflammatory networks interactions, and the multitude of discovered -omics without assignment demands that order must rule. Organ-based physiology supported by functional measurement and patient monitoring is the common language in medicine [33].
5. How should Systems Medicine proceed from the research bench forward? A perpetual loop must be established from bench to medical practice to bench and back again. It is important to establish the organizing principles and hierarchical multi-level design in Systems Medicine. End users in this process are both bench researchers and clinical physicians and their patients. The realities of bedside medicine should drive research efforts as much as the laboratory brings exciting new discoveries forward to benefit the patient.
6. Is there a summary of goals for Systems Medicine? Yes, the formulation of hierarchical models and guiding principles. The models and principles must organize forthcoming information and reduce the inherent complexity—be coherent, achieve clarity, and communicate cause and effect in a simplified, understood fashion [122, 123].

## Conclusion

Medicine is at a major transition point in history. Systems Theory offers organizing principles, holism, and a framework for emergent properties to form the basis for Systems Medicine. Maintaining complexity within at the leading edge of science, Systems Medicine must provide simplicity without for the human.
